# Patterns of Care in Patients with Basilar Artery Occlusion (BAO): A Population-Based Study

**DOI:** 10.3390/life13030829

**Published:** 2023-03-19

**Authors:** Mudassir Farooqui, Asad Ikram, Sajid Suriya, Fares Qeadan, Piotr Bzdyra, Syed A. Quadri, Atif Zafar

**Affiliations:** 1 Department of Neurology, University of Iowa Hospitals and Clinics, Iowa City, IA 52242, USA; 2 Department of Neurology, Beth Israel Deaconess Medical Center, Boston, MA 02215, USA; 3 Department of Neurology, University of New Mexico Health Science Center, Albuquerque, NM 87106, USA; 4 Department of Family and Preventive Medicine, University of Utah, Salt Lake City, UT 84112, USA; 5 Department of Neurology, St. Bernardine Medical Center, San Bernadino, CA 92404, USA; 6 Department of Neurology, University of Cincinnati, Cincinnati, OH 45221, USA; 7 Department of Neurology, St. Michael Hospital, University of Toronto, Toronto, ON M5B 1W8, Canada

**Keywords:** basilar artery occlusion, stroke, mechanical thrombectomy, posterior circulation stroke

## Abstract

Basilar artery occlusion (BAO) is associated with high morbidity and mortality. Endovascular therapy (EVT) has been shown to be beneficial in acute BAO patients. This retrospective observational study used the National Inpatient Sample (NIS) database to identify BAO patients using the International Classification of Diseases (ICD). Multivariable models were used to evaluate the association of risk factors, comorbidities, length of stay (LOS) in hospital, total cost, disposition, and transfer status. A total of 1120 (447 females, 39.95%) patients were identified, with a higher proportion of White individuals (66.8% vs. 57.6%), atrial fibrillation (31.5% vs. 17.2%; *p <* 0.0001), and peripheral vascular disease (21.2% vs. 13.7%; *p* = 0.009). A lower proportion of individuals with diabetes mellitus (32.1% vs. 39.5%; *p* = 0.05) was found in the EVT group. Majority of the patients (924/1120, 82.5%) were treated at the urban teaching facility, which also performed most of the EVT procedures (164, 89.13%), followed by non-academic urban (166, 14.8%) and rural (30, 2.7%) hospitals. Most patients (19/30, 63%) admitted to rural hospitals were transferred to other facilities. Urban academic hospitals also had the highest median LOS (8.9 days), cost of hospitalization (USD 117,261), and disposition to home (32.6%). This study observed distinct patterns and geographical disparities in the acute treatment of BAO patients. There is a need for national- and state-level strategies to improve access to stroke care.

## 1. Introduction

Acute ischemic stroke (AIS) is one of the leading causes of mortality and long-term disability. Each year, in the United States (US), nearly 800,000 people are diagnosed with AIS [1,2]. It is estimated that AIS is associated with approximately USD 50 billion in annual healthcare spending [1,2]. In 2015, after the pivotal trials, endovascular thrombectomy (EVT) was established as a safe and effective treatment for the AIS due to anterior circulation large vessel occlusion (LVO) strokes [1]. Results from these trials consistently showed that EVT results in better functional outcomes and leads to a substantial improvement in quality of life, thereby establishing EVT as the standard of care by the American Heart Association/American Stroke Association (AHA/ASA) [1].

Basilar artery occlusion (BAO) is an infrequent cause of AIS, representing approximately 3–5% of all strokes [3]. Despite recent advancements in stroke treatment and management, BAO is associated with poor clinical outcomes whereby patients with severe symptoms may either die or become functionally dependent [4,5,6]. Acute management of BAO involve the use of anticoagulants, intravenous and/or intra-arterial thrombolysis (alteplase), and endovascular thrombectomy (EVT) [3,7]. Intravenous tissue plasminogen activator (IV-tPA) is considered the preferred treatment for acute BAO; however, several studies have observed inadequate rates of successful reperfusion with IV-tPA, resulting in insufficient clinical outcomes and poor disease sequelae [8,9]. On the other hand, multiple prospective reports from registries have demonstrated that EVT in BAO patients results in better successful reperfusion, with favorable outcomes. However, the effectiveness of both IV-tPA and EVT therapy decreases with delayed administration relative to symptom onset [10,11].

Stroke treatment is highly time-sensitive. Factors such as stroke severity, comorbid conditions, timely triage and treatment initiation, hospital stay, and use of reperfusion therapies play important roles in optimal stroke recovery [1,3]. The stroke care model is facilitated via a coordinated hospital network, where patients are triaged and evaluated in the field and directed to the nearest stroke center. These patients can either be transported to the nearest stroke center for evaluation and IV-tPA therapy or can be directly taken to an EVT-capable center for both IV-tPA and EVT, when LVO is suspected. However, this triage system has lagged behind the emerging available treatments. Although stroke patients triaged to a non-EVT-capable center maybe provided with fast access to IV-tPA, it may delay the initiation of EVT in eligible patients, thus depriving the patients of an effective treatment. On the contrary, in the US, approximately 37% of stroke centers have the capability to provide EVT, but <25% of the US population has access to these centers [12]. Moreover, triaging and transportation of patients from non-EVT-capable centers may result in substantial delays, which can lead to stroke progression, thereby attenuating the effects of EVT treatment and resulting in worsened functional outcomes and higher mortality rates [13,14]. Furthermore, these factors are enhanced by the disparity between urban and rural stroke care. Urban areas have relatively increased access to stroke centers providing IV-tPA and/or EVT therapy, hence decreasing the time to treatment as compared to the rural population [15,16].

Although EVT has recently been shown to be beneficial, there exists a geographical disparity in its clinical utilization in acute BAO patients. In this study, we aim to describe factors and temporal trends of EVT and explore determinants of treatment impact, including length of stay (LOS) in hospital, discharge status, and cost of care in patients with AIS due to BAO.

## 2. Materials and Methods

### 2.1. Data Source and Study Design

This is a retrospective, observational cohort study utilizing data from the 2015 National Inpatient Sample (NIS) database. The NIS is part of the Healthcare Cost and Utilization Project (HCUP) developed by the Agency for Healthcare Research and Quality. It is the largest all-payer US inpatient database comprising of participating non-federal and community hospitals across 49 states with approximately 5–8 million hospital stays each year [17]. The NIS database comprises information related to inpatient hospital stays and is used for evaluating national trends in healthcare, disease burden, cost, and quality. The dataset comprises clinical and nonclinical variables associated with hospital stays, including primary and secondary diagnoses, and procedures, patients’ admission and discharge statuses, and patients’ demographic information. The NIS database is publicly available at http://www.hcup-us.ahrq.gov. All the analyses complied with the NIS data use agreement, and the study was exempt from the Institutional Review Board (IRB) due to the de-identified nature of publicly available data. The study is reported in accordance with the Strengthening the Reporting of Observational Studies in Epidemiology statement [18].

### 2.2. Patients’ Characteristics and Data Elements

We used the International Classification of Diseases, Ninth Revision, Clinical Modification (ICD-9-CM) codes to identify the diagnoses of basilar artery occlusion (433.01). Patients who underwent endovascular thrombectomy treatment were identified by procedure code (39.74). The patients’ baseline characteristics, including demographics, age, gender, vascular risk factors and comorbidities, social habits (e.g., alcohol and tobacco exposure, drug abuse), and medical complications were extracted. Clinical variables included hospital characteristics, location/status of the hospital (academic, urban non-academic, or rural), length of stay (LOS), hospital charges, in-hospital mortality, transfer, and disposition of the patient.

### 2.3. Statistical Analysis

Continuous variables were represented by the mean and standard deviation (SD), and categorical variables as numbers and percentages. Normality of distribution was assessed using histograms and the Shapiro–Wilk test. Student’s t-tests or the Wilcoxon rank-sum test was used for univariate analysis for continuous variables, and Chi-square or Fisher’s exact tests for categorical variables. Multivariable regression models with complex designs were utilized to examine associations between the variables, including LOS, total charges, disposition, and transfer status. For all the analyses, a *p*-value of <0.05 was considered statistically significant. All the analyses were performed using the SAS system, version 9.4 (SAS Institute Inc., Cary, NC, USA).

## 3. Results

A total of 1120 patients were identified with a diagnosis of BAO. The mean age of the cohort was 66 (±14.69) years, and 447 (39.95%) were female. The demographics and baseline characteristics of the EVT and non-EVT groups are shown in Table 1. There was a higher proportion of White individuals receiving EVT (66.8%). There was a higher proportion of patients with atrial fibrillation (31.5% vs. 17.2%; *p <* 0.0001) and peripheral vascular disease (21.2% vs. 13.7%; *p* = 0.009), whereas the proportion of diabetic patients was lower (32.1% vs. 39.5%; *p* = 0.05) in the EVT group as compared with the non-EVT group. Other risk factors were similar between the two groups (Table 1).

### Patient Flow and Hospital Characteristics:

**Figure 1:**
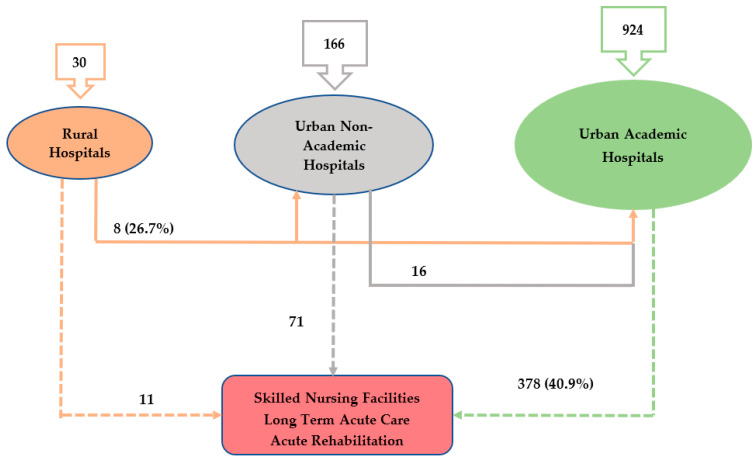
Transfer patterns of patients with BAO in different healthcare facilities during the study period.

The majority (924/1120, 82.5%) of the BAO patients were managed at the urban teaching facility, while 166 (14.8%) patients were admitted to non-academic urban centers and 30 (2.7%) to rural hospitals. Among the patients admitted to the rural hospitals, 8 (26.7%) were transferred to urban non-academic and academic hospitals, while 11 (36.6%) were transferred to other facilities (skilled nursing facility—SNF, long term acute care—LTAC, or acute rehabilitation—AR). Similarly, of the 166 patients admitted to the urban non-teaching hospitals, 16 (19.6%) were transferred to urban academic hospitals and 71 (42.8%) to other facilities (SNF, LTAC, or AR). Of the 924 (82.5%) patients admitted to urban teaching hospitals, 378 (40.9%) were transferred to other facilities (Table 1 and Figure 1). Moreover, a total of 184 patients underwent EVT procedures, of which 164 (89.13%) were performed in the urban teaching hospitals and 20 (11%) in the urban non-teaching hospital, while rural hospitals did not perform any EVT procedures.

**Figure 2:**
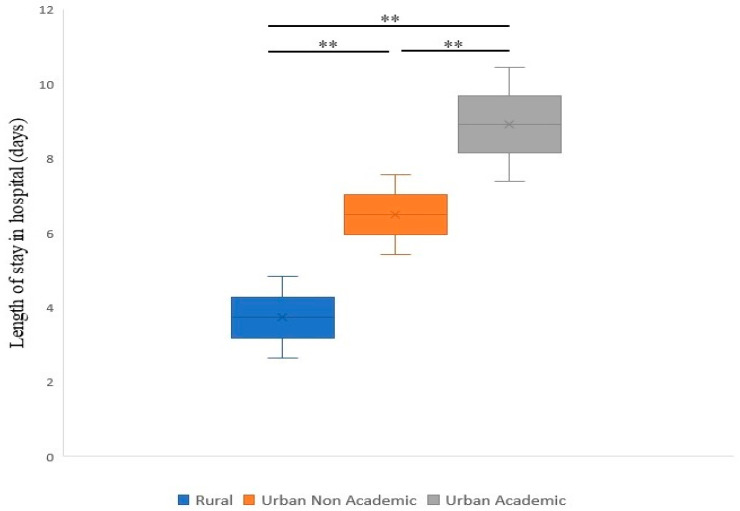
Median length of stay (LOS) in hospital for patients with BAO in different healthcare facilities (rural, urban non-academic, and urban academic) during the study period. ** *p* < 0.001.

**Figure 3:**
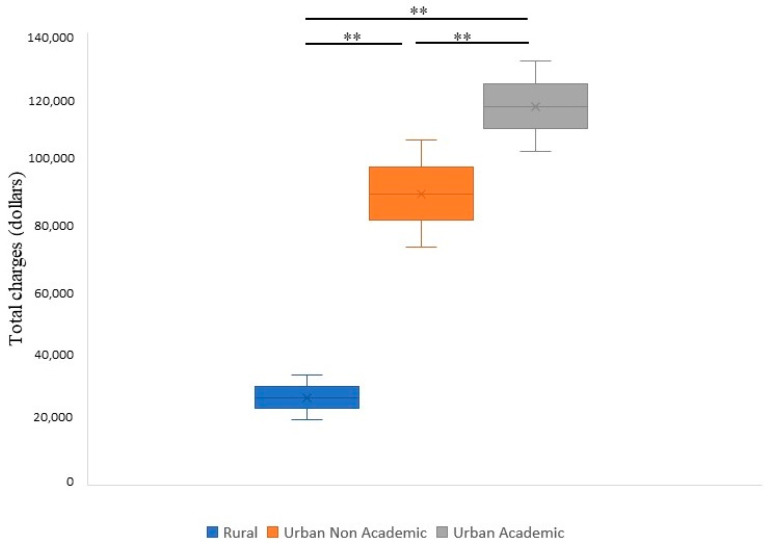
Total cost of hospitalization for patients with BAO in different healthcare facilities (rural, urban non-academic, and urban academic) during the study period. ** *p* < 0.001.

**Figure 4:**
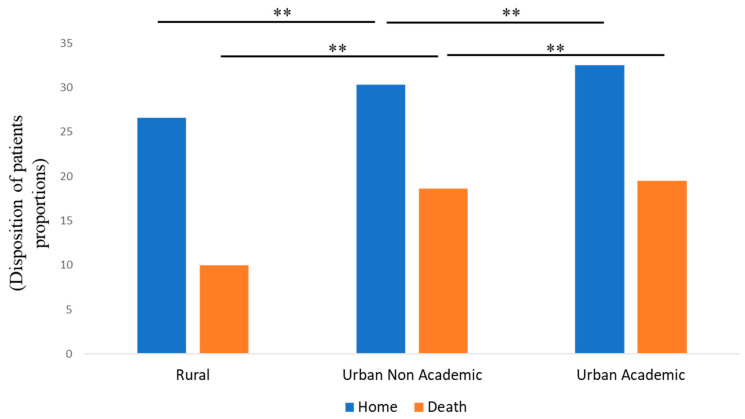
Disposition of patients with BAO from different healthcare facilities (rural, urban non-academic, and urban Academic) during the study period. ** *p* < 0.001.

A higher median length of stay (LOS) was noted for the BAO patients in urban academic centers (8.9 days), followed by non-academic urban centers (6.5 days) and rural hospitals (3.7 days) (Figure 2 and Table 2). Similarly, the stays with the highest median total costs were those of patients admitted to urban academic centers (USD 117,261), followed by urban non-academic (USD 90,148) and rural hospitals (USD 27,052) (Figure 3). Moreover, a higher proportion of patients were discharged home from urban academic centers (32.6%), followed by urban non-academic centers (30.34%) and rural hospitals (26.6%) (Figure 4 and Table 2). In-hospital mortality was 22.9% in the total cohort, with a higher proportion of patients in the EVT group (35.3%). Of these, 10% were noted in rural hospitals, 18.6% in non-teaching urban centers, and 19.5% in urban academic hospitals.

## 4. Discussion

In this population-based study, we found that the majority of the patients with acute BAO were managed at an urban academic hospital, which also performed most of the EVT procedures. We also observed that a significant proportion of patients admitted to the rural hospitals were transferred to other healthcare facilities. Moreover, the LOS and total cost of hospitalization were higher for the patients managed at urban hospitals.

Early recanalization has been shown to increase the reperfusion and functional outcomes in AIS patients. The current stroke care model incorporates pre-hospital triage and network hospitals along with timely identification and coordinated facilitation to provide prompt treatment. This stroke care model incorporates triage of the patients by emergency medical personnel and depends on the diagnostic accuracy for LVO in the field and the availability of treatment in nearby hospitals. In the drip and ship model, patients are first transported to the nearest local stroke center for evaluation and IV-tPA treatment, and, if eligible for the EVT, they are then transferred to the EVT-capable centers. Whereas, in the mothership model, these patients are directly transported to the EVT-capable centers, where they can be provided with IV-tPA and/or EVT therapy. 

Triage of these LVO patients to centers without EVT may result in significant delays in the initiation of EVT treatment; on the contrary, most of these patients, when transferred to an EVT-capable center, may become non-eligible for EVT treatment [14]. Conversely, if non-EVT-eligible stroke patients are directly transported to EVT-capable centers at the expense of travel time, it may deprive them of an early IV-tPA therapy at a nearby local stroke center. Therefore, the current AHA/ASA guidelines recommend incorporating the factors of stroke severity and EVT eligibility into prehospital stroke triage. Stroke guidelines recommend EVT for eligible patients in anterior circulation; however, there is a disparity in its clinical utilization in acute BAO patients. In our study, we observed that the rural centers did not perform any EVT procedures, as the majority of the procedures were performed in the academic hospitals. Moreover, a significant proportion of patients were transferred from rural centers to other acute care facilities, including both urban non-academic and academic hospitals. These findings of our study indicate a lack of EVT capabilities and/or multidisciplinary critical care services in rural hospitals, thereby resulting in these hospitals treating only mild/moderate stroke patients while transferring severe cases to other, more capable facilities. Recent reports have also observed that approximately one-third of the stroke centers in US have the capability to provide EVT; 30% of the US population has access to an EVT center within 30 min, and nearly 50% can access it within 60 min [12,19]. Moreover, studies have also demonstrated that patients who are first transported to nearby stroke centers and then transferred to EVT-capable centers may experience significant delays in EVT treatment, resulting in worsened functional outcomes [14]. However, in our current study, we were not able to evaluate the transfer times for patients since this publicly available database does not allow the identification of hospital metrics. These results thus confer findings from studies in anterior circulation stroke patients.

There is a dire need to optimize the treatment paradigm of the stroke care model. The current stroke triage system largely employs the drip-and-ship paradigm, whereby stroke patients with high suspicion of an LVO are directed to receive the IV-tPA at the earliest time point. However, this model is associated with significant delays in instituting EVT in eligible patients, as the interhospital transfer process results in significant time constraints [14]. Several factors that may contribute to this treatment dilemma include prehospital inaccuracy of the stroke diagnosis, inefficient in-hospital protocols, and lack of availability of efficient interhospital transportation, especially when IV-tPA therapy has been instituted. However, recently, there has been increasing interest in facilitating the accessibility of EVT procedures to eligible patients, which involves expanding EVT services to rural areas, using mobile stroke units (MSUs) for triage and treatment, and employing mobile interventional stroke teams or a pre-hospital triage system for the timely transport of eligible patients to EVT-capable centers [12,20].

Our study also observed that a higher proportion of males (61.4%) and White individuals (66.8%) were treated with EVT. Moreover, we also observed a higher proportion of patients with atrial fibrillation (AF) (31.5%) and a lower proportion with diabetes mellitus (39.5%) in the EVT group as compared with the non-EVT group. These observations are reflective of the general practice and are in line with the previous findings, which reported that higher blood glucose levels are associated with decreased rates of recanalization, functional outcome, and mortality [21,22,23,24]. Consistent with our findings, other studies have also reported a higher proportion of patients with AF undergoing EVT, probably because cardiac emboli frequently cause BAO [3]. Moreover, some studies have also reported AF to be an independent predictor of an increased rate of successful recanalization in BAO patients [25,26], whereas other studies have not reported any association of AF with functional or procedural outcomes [27,28].

Our study also observed that these acute BAO patients treated at urban academic hospitals had the highest median LOS and cost of hospitalization as compared to those treated at rural and urban non-academic centers. This is most likely because these patients had a more severe disease, although we were not able to evaluate this since the NIS database lacks information on stroke severity. These findings also corroborate with the increased number of EVT procedures being performed by these centers. Hence, the increased cost of hospitalization in urban academic hospitals may be reflective of an increased number of EVT procedures and/or the use of multi-disciplinary specialty and critical care services. Moreover, it was also observed that these urban academic hospitals had higher mortality rates as compared to non-academic and rural centers. This may be a result of delayed initiation of treatment because of the in-field triage and transportation delays, ease of access to EVT-capable centers, transfer of more severe patients, and/or delays in inter-hospital transfer [14,29]. On the other hand, there was a higher proportion of patients discharged home from urban academic centers, indicating an effective treatment and management, although at the expense of a higher cost of care.

In recent years, EVT has been established as the standard of care for AIS in anterior circulation [30,31,32,33,34,35,36]. Studies from prospective registries have also observed beneficial effects of EVT in the procedural and functional outcomes of strokes due to BAO [37,38]. Two previously published randomized trials, BEST (Basilar Artery Occlusion Endovascular Intervention Versus Standard Medical Treatment) and BASIC (Basilar Artery International Cooperation Study), comparing EVT versus the best medical treatment, were inconclusive in establishing the efficacy of EVT treatment in BAO patients [5,6]. Although these trials failed to demonstrate statistically significant differences in primary favorable clinical outcomes, there was a trend observed in the secondary analysis towards good clinical outcomes (modified Rankin Scale 0–3) with EVT among these acute BAO patients. However, both trials were subjected to a lack of timely enrollment, with an increased number of patients being treated outside the trial and a high crossover rate, which may have led to potential bias [39]. These trials were followed by two recently concluded studies, Basilar Artery Occlusion Chinese Endovascular (BAOCHE) trial and Endovascular Treatment for Acute Basilar Artery Occlusion (ATTENTION) [40,41]. Both trials demonstrated the efficacy of EVT in BAO patients. The results showed that EVT was associated with a significant increase in the primary endpoint of good clinical outcome (mRS 0–3) at 90 days in the EVT group (BAOCHE: aRR: 1.81, 95% CI: 1.26–2.6, *p <* 0.001, NNT: 4.5 and ATTENTION: RR: 2.06, 95% CI: 1.46–2.91, *p <* 0.001, NNT: 4) when compared to the medical management [40,41]. Moreover, there was a significant trend towards higher symptomatic intracranial hemorrhage (sICH) and lower mortality in the EVT groups when compared to the medical management. Thus, providing the evidence that EVT leads to better functional outcomes in patients with BAO when compared with the best medical management [5,6,40,41].

Moreover, since the pivotal EVT trials, there has been an increase in strategies to improve the accessibility of EVT. However, population density, resource allocation, and geographical location (urban versus rural) are some of the major limiting factors in optimizing the stroke care treatment [12,19,42]. Various strategies have been employed to increase the access of thrombectomy. There has been an increase in stroke education programs emphasizing the need for early recognition and prompt action. Pre-hospital in-field screening scores for LVO detection have demonstrated to be an effective tool for stroke triage, with several scales being implemented by local EMS personnel [43,44,45,46,47]. Telestroke services are increasingly being used to facilitate the determination of treatment eligibility. Mobile stroke units have also been shown to improve the prehospital stroke triage process by expediting stroke treatment and may help in early identification of potentially eligible patients, resulting in a direct transfer to an EVT-capable center. The trip and treat strategy have also been employed to facilitate the EVT access by enabling travel to a non-EVT-capable center to perform the procedure. More importantly, there has been a recent increase in advocacy to expand EVT access by increasing the number of EVT-capable centers. With the recent advancements in stroke care, it is essential to identify gaps in the healthcare system and tailor solutions to improve access to care.

In summary, our study provided information on the impact of systems of care, identifying urban–rural disparities in the treatment paradigm for patients with acute BAO. These results are reflective of the access constraints in the current healthcare system. While there is a need to expand the access of care, an important challenge is the population and geographical heterogeneity. Moreover, strategies to improve the efficiency of the existing system should be an area of focus. Therefore, it is imperative that the national and regional policies prioritize the availability of resources to improve access to EVT to mitigate the healthcare disparity.

### Strengths and Limitations

The NIS database provides a representation of the US population across various hospital settings (urban versus rural), thus providing a capacity to study this uncommon stroke condition. However, the study has inherent limitations regarding the use of administrative data, including documentation and coding variation across different hospitals, which can potentially result in underestimation. Additionally, it does not contain information on patient-level data such as diagnostic and imaging studies, stroke severity (National Institutes of Health Stroke Scale—NIHSS), functional status (modified Rankin Scale—mRS), procedural characteristics (modified Treatment in Cerebral Ischemia—mTICI), or stroke workflow time metrics (time to needle, groin puncture, etc.). These variables are significant predictors and relevant confounding factors that impact the outcome; however, the limitations of the data did not allow us to analyze sub-groups based on stroke severity or functional outcomes. Despite these limitations, this study provides valuable information on the national trends and factors associated with stroke care in acute BAO patients.

## 5. Conclusions

This study evaluated patterns of care in patients with stroke due to acute BAO. The majority of the patients were treated at an urban academic center, which also performed most of the EVT procedures. Rural hospitals transferred most of the patients to other healthcare facilities. These data demonstrate a national trend of acute stroke care in patients with BAO. There is a need for national- and state-level strategies to optimize the clinical and treatment paradigms and improve access to EVT for BAO stroke patients.

## Figures and Tables

**Table 1:** Demographics and clinical characteristics of patients with BAO.

	Total	EVT	Non-EVT	*p*-Value
	n (% )	n (% )	n (% )	
	1120 (100)	184 (16.43)	936 (83.57)	
**Demographics**				
Gender				0.6804
Male	672 (60.05)	113 (61.41)	559 (59.79)	
Female	447 (39.95)	71 (38.59)	376 (40.21)	
Age, mean ± SD	65.99 ± 14.69	64.74 ± 16.68	66.24 ± 15.32	0.2057
Race				0.0474
White	662 (59.11)	123 (66.85)	539 (57.59)	
Black	185 (16.52)	19 (10.33)	166 (17.74)	
Hispanic	85 (7.59)	15 (8.15)	70 (7.48)	
Others	92 (8.21)	10 (5.43)	82 (8.76)	
Unknown	96 (8.57)	17 (9.24)	79 (8.44)	
**Vascular Risk Factors (Comorbidities)**				
Diabetes				0.0519
Yes	429 (38.30)	59 (32.07)	370 (39.53)	
No	691 (61.70)	125 (67.93)	566 (60.47)	
Hypertension				0.1671
Yes	894 (79.82)	140 (76.09)	754 (80.56)	
No	226 (20.18)	44 (23.91)	182 (19.44)	
Atrial fibrillation				<0.0001
Yes	219 (19.55)	58 (31.52)	161 (17.20)	
No	901 (80.45)	126 (68.48)	775 (82.80)	
Congestive heart failure				0.3829
Yes	114 (10.18)	22 (11.96)	92 (9.83)	
No	1006 (89.82)	162 (88.40)	844 (90.17)	
Peripheral vascular disorders				
Yes	167 (14.91)	39 (21.20)	128 (13.68)	0.0088
No	953 (85.09)	145 (78.80)	808 (86.32)	
Coagulopathy				0.12
Yes	59 (5.27)	14 (7.61)	45 (4.81)	
No	1061 (94.73)	170 (92.39)	891 (95.19)	
Obstructive sleep apnea				0.153
Yes	61 (5.45)	6 (3.26)	55 (5.88)	
No	1059 (94.55)	178 (96.74)	881 (94.12)	
**Social Risk Factors**				
Alcohol				0.3088
Yes	67 (5.98)	14 (7.61)	53 (5.66)	
No	1053 (94.02)	170 (92.39)	883 (94.34)	
Tobacco exposure				0.5887
Yes	223 (20.80)	41 (22.28)	192 (20.51)	
No	887 (79.20)	143 (77.72)	744 (79.49)	
Drug Abuse				
Yes	59 (5.27)	6 (3.26)	53 (5.66)	0.1825
No	1061 (94.73)	178 (96.74)	883 (94.34)	
Location/teaching status of hospital				0.0091
Rural				
Urban /non-teaching	30 (2.68)	0 (0.00)	30 (3.21)	
Urban/teaching	166 (14.82)	20 (10.87)	146 (15.60)	
	924 (82.50)	164 (89.13)	760 (81.20)	
Died during hospitalization				<0.0001
Yes	256 (22.86)	65 (35.33)	191 (20.41)	
No	864 (77.14)	119 (64.67)	745 (79.59)	
Percutaneous endoscopic gastrostomy				<0.0001
(PEG) tube placement				
Yes	109 (9.73)	36 (19.57)	73 (7.80)	
No	1011 (90.27)	148 (80.43)	863 (92.20)	
Tracheotomy procedure				<0.0001
Yes	73 (6.52)	29 (15.76)	44 (4.70)	
No	1047 (93.48)	155 (84.24)	892 (95.30)	

EVT: endovascular thrombectomy.

**Table 2:** Multivariate logistic regression models.

Disposition Other than Home				Length of Stay		
	Odds Ratio	95% CI	*p*-Value	Odds Ratio	95% CI	*p*-Value
Age	1.033	0.256–1.058	0.462	1.45	−0.85–2.058	0.62
Sex	0.520	0.169–1.600	0.254	1.21	0.45–1.75	0.31
Cost	0.45	−2.45–1.43	0.598	1.67	0.76–3.87	0.23

## Data Availability

All the data will be made available from the corresponding author upon reasonable request.
